# Higher Abundance of Sediment Methanogens and Methanotrophs Do Not Predict the Atmospheric Methane and Carbon Dioxide Flows in Eutrophic Tropical Freshwater Reservoirs

**DOI:** 10.3389/fmicb.2021.647921

**Published:** 2021-03-17

**Authors:** Gabrielle Maria Fonseca Pierangeli, Mercia Regina Domingues, Tatiane Araujo de Jesus, Lúcia Helena Gomes Coelho, Werner Siegfried Hanisch, Marcelo Luiz Martins Pompêo, Flávia Talarico Saia, Gustavo Bueno Gregoracci, Roseli Frederigi Benassi

**Affiliations:** ^1^Institute of Marine Sciences, Federal University of São Paulo, Santos, Brazil; ^2^Center of Engineering, Modeling and Applied Social Sciences, Federal University of ABC, Santo André, Brazil; ^3^Chemical Engineering Department, Federal University of São Paulo, Diadema, Brazil; ^4^Ecology Department, State University of São Paulo, São Paulo, Brazil

**Keywords:** greenhouse gases, sediment microbiota, metagenomics, anthropic pollution, network analysis

## Abstract

Freshwater reservoirs emit greenhouse gases (GHGs) such as methane (CH_4_) and carbon dioxide (CO_2_), contributing to global warming, mainly when impacted by untreated sewage and other anthropogenic sources. These gases can be produced by microbial organic carbon decomposition, but little is known about the microbiota and its participation in GHG production and consumption in these environments. In this paper we analyzed the sediment microbiota of three eutrophic tropical urban freshwater reservoirs, in different seasons and evaluated the correlations between microorganisms and the atmospheric CH_4_ and CO_2_ flows, also correlating them to limnological variables. Our results showed that deeper water columns promote high methanogen abundance, with predominance of acetoclastic *Methanosaeta* spp. and hydrogenotrophs *Methanoregula* spp. and *Methanolinea* spp. The aerobic methanotrophic community was affected by dissolved total carbon (DTC) and was dominated by *Crenothrix* spp. However, both relative abundance of the total methanogenic and aerobic methanotrophic communities in sediments were uncoupled to CH_4_ and CO_2_ flows. Network based approach showed that fermentative microbiota, including *Leptolinea* spp. and *Longilinea* spp., which produces substrates for methanogenesis, influence CH_4_ flows and was favored by anthropogenic pollution, such as untreated sewage loads. Additionally, less polluted conditions favored probable anaerobic methanotrophs such as *Candidatus* Bathyarchaeota, *Sva0485*, *NC10*, and *MBG-D/DHVEG-1*, which promoted lower gaseous flows, confirming the importance of sanitation improvement to reduce these flows in tropical urban freshwater reservoirs and their local and global warming impact.

## Introduction

Carbon dioxide (CO_2_) and methane (CH_4_) are the main constituents of greenhouse gases (GHGs), representing 76 and 16% of total GHGs, respectively (Fagodiya et al., 2017). Anthropogenic emissions add up to 33.867.9 Tg CO_2_ Eq per year of CO_2_ and 11,849.5 Tg CO_2_ Eq per year of CH_4_ (Deemer et al., 2016). These gases are important sources of global warming, contributing to adverse effects on the economy, environment, and human health, such as loss of biodiversity and agricultural areas, and the increase of heart, respiratory, and infectious diseases (Franchini and Mannucci, 2015; Cramer et al., 2018). Among these GHGs, CH_4_ emissions are especially important, since they are approximately 84-fold more potent for the greenhouse effect than CO_2,_ over the first 20 years after their emission (Jackson et al., 2019). Previous studies have demonstrated that freshwater reservoirs emit CO_2_ and CH_4_ into the atmosphere (Gruca-Rokosz et al., 2011; Marcelino et al., 2015; Musenze et al., 2016; Gruca-Rokosz and Koszelnik, 2018), and the gaseous emissions increase when these environments are impacted by anthropic nutrient input, mainly in developing countries (Gonzalez-Valencia et al., 2014; Silva et al., 2016). Additionally, GHG emissions from freshwater reservoirs have a global importance, since the annual gaseous emission from these environments represent about 1.5% of equivalent global emissions of CO_2_ from anthropogenic sources, being compared with emissions from rice paddies and biomass burning, considering a period of 100 years (Deemer et al., 2016).

Both CO_2_ and CH_4_ can be produced from degradation of organic carbon by microorganisms (Schimel and Gulledge, 1998; Stams and Plugge, 2009; Wang et al., 2017), including a consortium of fermentative and acetogenic bacteria and methanogenic archaea in the anaerobic digestion process (Ferry, 2011). Thus, it is already known that microorganisms participate in climate change by producing CO_2_ and CH_4_, but they can also help mitigate it by consuming these gases (Cavicchioli et al., 2019). In freshwater reservoirs, the CO_2_ production occurs mainly in the water column (Gruca-Rokosz et al., 2011) by CH_4_ oxidation or organic matter respiration from local or allochthonous sources in the water body (Guérin et al., 2006). Higher microbial densities are correlated with GHG due to respiration (Anderson, 2016). However, processes such as photosynthesis, chemosynthesis and methanogenesis using CO_2_ consume the latter and reduce its emission (Casper et al., 2000; Wahid et al., 2019). On the other hand, CH_4_ in aquatic environments arises mainly from anaerobic sediments (redox potential under −200 mV) (Reeburgh and Heggie, 1977) with net flows driven by shallow water ebullition and deeper region diffusion (Gruca-Rokosz et al., 2011). Methanogenesis occurs through degradation of specific organic substrates by anaerobic archaea (Valentine, 2007), and is classified into hydrogenotrophic (using H_2_ and CO_2_ or formiate), acetoclastic (acetate) or methylotrophic (methyl groups) (Lyu et al., 2018). These substrates are provided by fermenters (generating acetate, formiate, CO_2_, H_2_, butyrate, and propionate) and acetogenics (converting the latter two into the former four compounds) (Ferry, 2011). Furthermore, methanotrophic microorganisms can consume CH_4_, which is oxidized prior to atmospheric release, reducing its flow and global warming impact (Zigah et al., 2015; Martinez-Cruz et al., 2018). It is known that CH_4_ solubility and a well established methanotrophic community are important factors to control CH_4_ emission in aquatic ecosystems (Wen et al., 2018; Valenzuela et al., 2020). This methanotrophic community can include aerobic methanotrophic bacteria or anaerobic methanotrophic archaea (ANME), the last one participating of CH_4_ oxidation by reversion of the methanogenic pathway (Timmers et al., 2017) and by association with bacteria (Su et al., 2020). This interaction between archaeal and bacterial domains for CH_4_ oxidation is coupled to the reduction of compounds such as sulfates or nitrates/nitrites, and elements such as metallic ions, removing them from the environment (Cui et al., 2015; Kiran et al., 2015; Naqvi et al., 2018). Besides these, some methanogens seem able to oxidize CH_4_ to CO_2_ as well (Arshad et al., 2015; Cai et al., 2018).

In aquatic environments, limnological conditions of bottom water, such as oxygen concentration, organic matter availability, temperature and water depth may also influence methanogenesis and/or CH_4_ oxidation, affecting gas flows (West et al., 2016; Chronopoulou et al., 2017; Maltby et al., 2018). However, little is known about the microbiota and the environmental factors that promote gaseous flows in tropical urban reservoirs (Musenze et al., 2016), and there is a lack of information for environmental factors and mediating microbiota associated with gaseous flows both temporally and within/across these ecosystems. Additionally, given the potential of freshwater reservoirs to promote CH_4_ and CO_2_ flows and microbial participation in producing and consuming these gases, knowledge of microbial communities will allow a better understanding of gaseous dynamics in these ecosystems. In the present study, we addressed several gaps in the current knowledge of environmental factors that drive the microbiota acting in CH_4_ and CO_2_ flows in urban tropical freshwater reservoirs impacted by anthropogenic activities. We studied spatially the sediment microbiota and limnological variables in three eutrophic urban tropical freshwater reservoirs in five seasons from 2018 to 2019 and verified the correlations among sediment bacteria and archaea, CH_4_ and CO_2_ flows and limnological variables in these reservoirs. To the best of our knowledge this is the first study that accesses spatially and seasonally the environmental key factors that drive microbiota mediating methane and carbon dioxide fluxes in eutrophic tropical urban reservoirs. We applied cutting edge tools to analyze the data improving our understanding and opening up possibilities to minimize the emission of these GHG.

## Materials and Methods

### Study Area and Sampling

**FIGURE 1:**
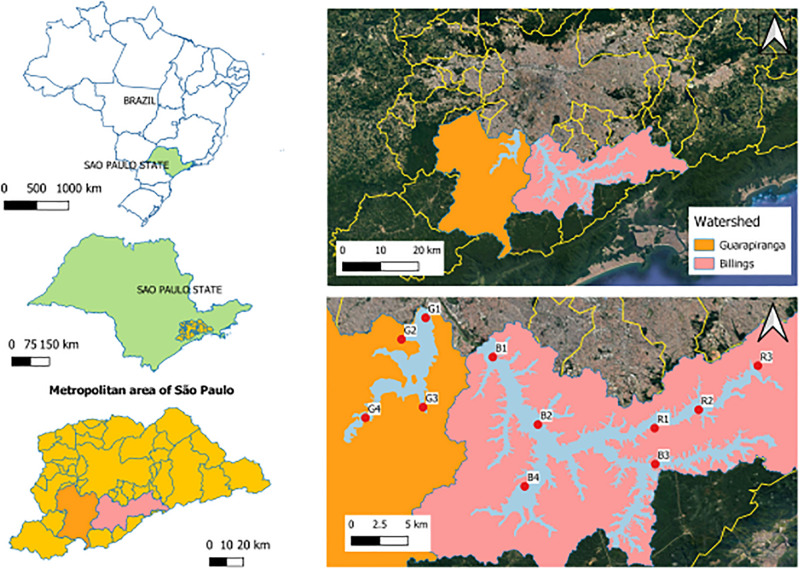
Study area in São Paulo, Brazil. Highlight to the sampling stations in: Guarapiranga (G1–G4), Billings (B1–B4), and Rio Grande (R1–R3) reservoirs. Source: Google/Satellites.

The study area comprised the Guarapiranga (23°40′S, 46°43′), Billings (23°47′S, 46°35′W) and Rio Grande (23°45′S, 46°31′W) reservoirs, in the São Paulo Metropolitan Area (São Paulo, Brazil), an area with notorious importance since their main function is water supply (Pires et al., 2015). These three tropical freshwater reservoirs are eutrophic, due to the discharge of raw or inefficiently treated sewage (Cardoso-Silva et al., 2014, 2017). In each reservoir there were five sampling campaigns throughout 1 year, covering five hydrological periods: two wet seasons (summer Feb/2018 and Jan/2019), a dry season (winter Aug/2018) and two intermediate seasons (autumn May/2018 and spring Nov/2018). All gas, water and sediment samplings were performed *in situ*, with locations reached by small boats in two subsequent days in each period. There were eleven sampling stations (Figure 1), Guarapiranga (G1, G2, G3, and G4), Billings (B1, B2, B3, and B4), and Rio Grande (R1, R2, and R3) selected according to a compartmentalization pattern proposed beforehand (Cardoso-Silva et al., 2014, 2017).

In the Guarapiranga reservoir, G1 (23°40′49″S, 46°43′26″W) has intense urbanization in the surroundings, with possible input of urban effluents; G2 (23°41′56″S, 46°44′41″W) is an area with critical water quality conditions; G3 (23°45′27″S, 46°43′34″W) is a hypereutrophic area that receives the transpositions of B4 waters and with the worst water quality conditions due to the anthropogenic impacts; and G4 (23°45′60″S, 46°46′33″W) is a dendritc area with low urbanization (Cardoso-Silva et al., 2017). In the Billings reservoir, B1 (23°42′51″S, 46°39′57″W) is a mesotrophic area; B2 (23°46′21″S, 46°37′37″W) has untreated sewage discharge and high urbanization in the surrounding; B3 (23°48′24″S, 46°31′32″W) is a dendritic area with low urbanization and better water quality; and B4 (23°49′33″S, 46°38′18″W) also receives raw sewage discharge. In the Rio Grande reservoir, R1 (23°46′32″S, 46°31′34″W) is close to a water abstraction for human supply and receives constant applications of algaecides, such as hydrogen peroxide and copper sulfate; R2 (23°45′35″S, 46°29′17″W) is an eutrophic area; and R3 (23°43′18″S, 46°26′13″W) is also an eutrophic area with possible input of clandestine effluents from irregular urbanization in the surroundings, in addition to domestic sewage discharge (Cardoso-Silva et al., 2014).

### Water and Sediment Samplings and Limnological Measurements

Bottom water was measured *in situ* through a multiparametric probe Hydrolab DS5X (Hach), including temperature, electrical conductivity (EC), pH, dissolved oxygen (DO), ammonium, and nitrate. Bottom water samples were collected in a five-liter horizontal Van Dorn bottle (Limnotec), transferred in triplicate to decontaminated flasks (HCl 10% V/V wash) and transported in coolers to the laboratory. Analyses included dissolved total carbon (DTC), dissolved inorganic carbon (DIC), dissolved organic carbon (DOC), phosphorus (P), orthophosphate (P-PO_4_^3–^), total nitrogen (TN), total suspended solids (TSS), and biochemical oxygen demand (BOD) through standard methodologies (Baird et al., 2017).

Superficial sediment samples (0–20 cm) were collected with a manual Van Veen dredge, in triplicate, stored in sterilized recipients, and transported in coolers to the laboratory, where total phosphorus concentrations were determined (Andersen, 1976), along with carbon, hydrogen, nitrogen and sulfur contents in an automatic elemental analyzer Flash EA 1112 (Shimadzu).

### CH_4_ and CO_2_ Samplings and Flow Determinations in the Air-Water Interface

Gas sampling was performed with floating chambers adapted from field static chamber (Environment Agency, 2010) in which the gaseous matrix volume was sampled in triplicate, in intervals of 5, 10, and 15 min, stored at room temperature, and transported to the laboratory. The CH_4_ and CO_2_ concentrations were determined in mg m^2^ h^–1^, through Gas Chromatography–Flame Ionization Detector (GC-FID, Varian CP 8400, United States) (Yuesi and Yinghong, 2003), and converted to gas flow rate express in mg m^–2^ d^–1^, calculated from emission rates of the compounds in the static chamber, from the rate of change in the species concentration determined by GC-FID and with sampling intervals in the chamber using the first Fick’s law, as follows (Venterea, 2010):


$$F=\rho \,\left(\frac{V}{A}\right)\,\left(\frac{d\,C}{d\,t}\right)$$

Where: *F* is the flow of gases on the water surface (mg m^–2^ d^–1^); ρ is the specific mass of the air according to the temperature during the sampling (mg m^–3^); *V* is the internal volume of the chamber (m^3^); *A* is the surface area of the chamber exposed to water (m^2^); and (dC/dt) is the concentration gradient of the target species as a function of sampling intervals. The estimated CH_4_ flow rate limit of detection was 120 mg m^–2^ d^–1^.

### Sediment DNA Extraction and Quantitation

Sediment samples for DNA extraction were subsampled, washed thrice with phosphate-buffered saline (130 mmol L^–1^ NaCl, 7 mmol L^–1^ Na_2_HPO_4_, 3 mmol L^–1^ NaH_2_PO_4_, and pH 8.0) and received an equal part of Tris-EDTA (100 mmol L^–1^ Tris-Cl, 50 mmol L^–1^ EDTA, pH 8.0). The DNA extraction was conducted with adaptations of CTAB protocol (Minas et al., 2011). Samples received CTAB buffer (50 mmol L^–1^ Tris-Cl, 25 mmol L^–1^ EDTA, pH 8.0, 1% CTAB, 0.7 mol L^–1^ NaCl) and proteinase K and were incubated at 55°C in a water bath for 2 h. Samples were washed sequentially twice with phenol:chloroform:isoamyl alcohol (25:24:1) and once with chloroform. DNA was precipitated with NaCl and ethanol overnight at 4°C, washed with 70% ethanol, air dried, and eluted in milli-Q water overnight at 4°C. Extracted DNA samples were quantified by absorbance in a NanoDrop 2000 equipment (Thermo Scientific).

### 16S rDNA Amplification and Sequencing

The hypervariable v3-v4 region of the 16S rDNA gene was amplified through PCR, whose reactions followed the manufacturer’s protocol (PCR Master Mix 2x–Thermo Fisher) using 20 ng μL^–1^ final concentration of DNA and primers 341F (5′-TCGTCG GCAGCGTCAGATGTGTATAAGAGACAGCCTACGGGNGGC WGCAG) and 805R (5′-GTCTCGTGGGCTCGGAGATGTGTAT AAGAGACAGGACTACHVGGGTATCTAATCC) (Zheng et al., 2015) with sequencing adapters as recommended by Illumina MiSeq protocol. The amplification conditions in the Veriti thermal cycler (Applied Biosystems) were as follow (Ngoune et al., 2019): 95°C for 5 min, 30 cycles of 95°C for 30 s, 50°C for 45 s and 72°C for 1 min 30 s, and a final step of 72°C for 10 min. Amplicons were verified through agarose gel electrophoresis. Each sample was amplified in triplicate, which were mixed for Illumina MiSeq sequencing, in a 2 × 250 bp paired end run according to the manufacturer’s protocols.

### Bioinformatics and Statistics

Sequences were quality filtered with SolexaQA + + using the *dynamictrim* option (Cox et al., 2010). Sequences under 75 bp were removed with homemade Perl script (available on request), and paired reads were joined with PANDAseq (Masella et al., 2012). Sequences containing undetermined bases were also removed (Sinclair et al., 2015). Chimera were checked in random samples using CATCh (Mysara et al., 2015), and self-reference. OTUs were clustered with Swarm (Mahé et al., 2014), and Mothur was used for classification (Schloss et al., 2009), against Silva v1.32 (Quast et al., 2013). Classified reads were further used for metabolic inference with the FAPROTAX database (Louca et al., 2016).

The relative abundance of the total methanogenic and methanotrophic communities at genus level, the gaseous flows and the limnological variables were statistically analyzed adopting the Kruskal–Wallis test using PAST v4.03 (Harper and Ryan, 2001), and Scott–Knott test using SISVAR v5.7 (Ferreira, 2011) and compared according to seasons and sampling stations. Sediment archaea and bacteria acting in air-water gaseous flows were identified by correlating them with CH_4_ and CO_2_ flows, using the R package WGCNA v1.69 (Langfelder and Horvath, 2008). In this analysis, the limnological variables were also included. Rare genera (counts under five in at least 20% of samples) were removed and the remainder was Hellinger transformed with R package vegan v2.5-6 (Oksanen et al., 2019). Limnological variables and CH_4_ and CO_2_ flows were Z-score standardized in this step, given their different scales. We used an *R*^2^ > 0.7 and a soft threshold of 5, selecting modules with a minimum size of 60. Microorganisms correlated with CH_4_ and CO_2_ (*p*-value under 0.05) were explored in networks with a threshold of 0.15, using Cytoscape v3.7.2 (Shannon et al., 2003).

## Results

### Relative Abundance of the Total Methanogens and Aerobic Methanotrophs, Gaseous Flows and Limnological Variables

**FIGURE 2:**
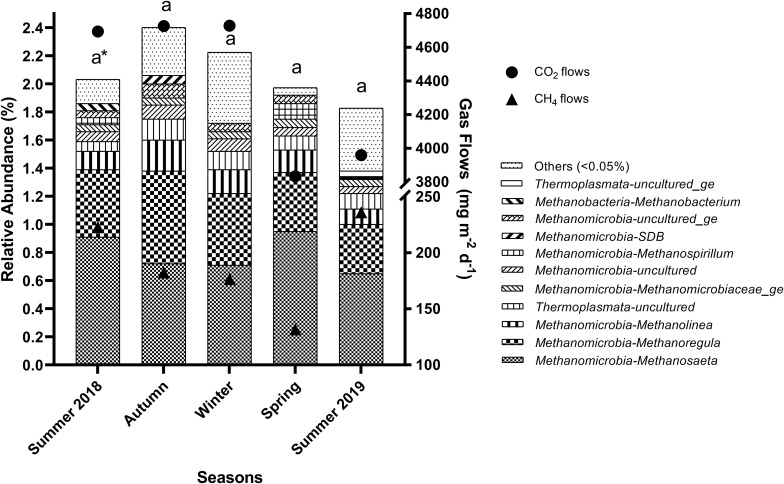
Relative abundance of methanogenic archaea genera in the sediments of reservoirs and the atmospheric CO_2_ and CH_4_ flows throughout the seasons. “Others” refers to a group of genera with abundances lower than 0.05%. *Means with equal letters do not differ by the Scott–Knott test at 5% significance to methanogens abundance.

**FIGURE 3:**
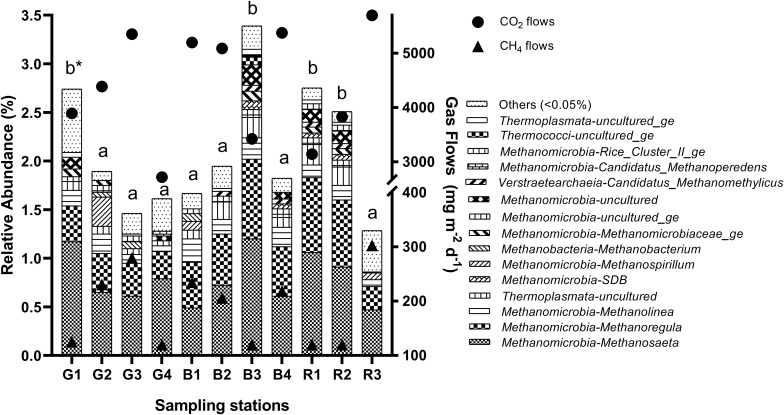
Relative abundance of methanogenic archaea genera in the sediment of reservoirs and the atmospheric CO_2_ and CH_4_ flows throughout the sampling stations. “Others” refers to a group of genera with abundances lower than 0.05%. *Means with equal letters do not differ by the Scott–Knott test at 5% significance to methanogens abundance.

Several methanogenic archaea genera (abundance > 0.05%) were present in reservoirs throughout the seasons (Figure 2) and stations (Figure 3). *Methanosaeta* spp. was the most abundant methanogen in all seasons and stations, followed by *Methanoregula* and *Methanolinea* spp. all from the *Methanomicrobia* class. Among the seasons, the relative abundance of the total methanogenic community was similar (Figure 2), according to Kruskal–Wallis and Scott–Knott tests. Nevertheless, these tests showed that temperature (*p* = 1.0e-07) was naturally higher in both summer 2018 (24.6 ± 0.9°C) and summer 2019 (25.0 ± 1.1°C), and lower in winter (19.4 ± 1.7°C) (Supplementary Table 1). Additionally, several other limnological variables also presented statistical differences among the seasons (Supplementary Table 1), including sediment N (*p* = 0.0168), C (*p* = 0.0038), H (*p* = 0.0006), S (*p* = 0.0145), P (*p* = 0.0011), water P (*p* = 0.0491), P-PO_4_^3–^ (*p* = 0.0038), DOC (*p* = 0.0288), pH (*p* = 0.0044), nitrate (*p* = 2.0e-06), ammonium (*p* = 0.0004), TN (*p* = 0.0029), and TSS (*p* = 0.0084), but no the CH_4_ and CO_2_ flows.

The relative abundance of the total methanogenic community changed spatially (*p* = 0.0168), and was higher in G1 (2.74%), B3 (3.40%), R1 (2.77%), and R2 (2.50%) (Figure 3). Similarly, depths differed among sampling stations (*p* = 3.4e-05), and were higher at the sites mentioned above, G1 (8.8 ± 1.3 m), B3 (10.1 ± 1.1 m), R1 (9.0 ± 0.9 m), and R2 (9.40 ± 2.1 m), but also in B1 (8.2 ± 1.5 m) (Supplementary Table 2). Additionally, CH_4_ (*p* = 0.0002) and CO_2_ (*p* = 0.0001) flows (Supplementary Table 2 and Figure 2) presented differences among the sampling stations. For CH_4_, the lowest flow rates occurred in G1 (125.2 ± 9.1 mg m^–2^ d^–1^), G4, B3, R1, and R2 (<120 mg m^–2^ d^–1^). Similarly, the lowest CO_2_ flows occurred also in G1 (3,895.1 ± 729.8 mg m^–2^ d^–1^), G4 (2,718.2 ± 625.3 mg m^–2^ d^–1^), B3 (3,423.6 ± 426.2 mg m^–2^ d^–1^), R1 (3,141.6 ± 387.2 mg m^–2^ d^–1^) and R2 (3,829.9 ± 282.8 mg m^–2^ d^–1^). Concerning the seasonal pattern, several differences were noted in limnological variables among sampling stations, such as sediment C content (*p* = 0.0131), water P (*p* = 0.0453), DTC (*p* = 0.0023), DIC (*p* = 0.0009), DOC (*p* = 0.0341), DO (*p* = 0.0436), and EC (*p* = 3.3e-05), but none of these differences occurred between G1, B3, R1, R2, and the other stations, not reflecting on the relative abundance of the total methanogenic community.

**FIGURE 4:**
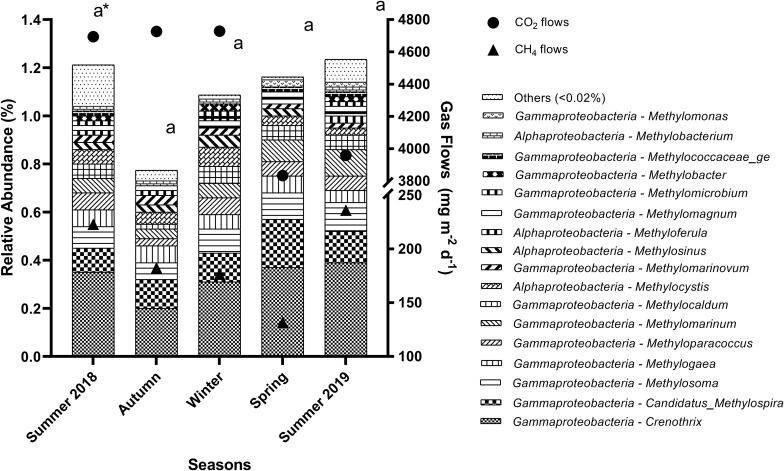
Relative abundance of aerobic methanotrophic genera in the sediment of reservoirs and the atmospheric CO_2_ and CH_4_ flows throughout the seasons. “Others” refers to a group of genera with abundances lower than 0.02%. *Means with equal letters do not differ by the Scott–Knott test at 5% significance to methanotrophs abundance.

**FIGURE 5:**
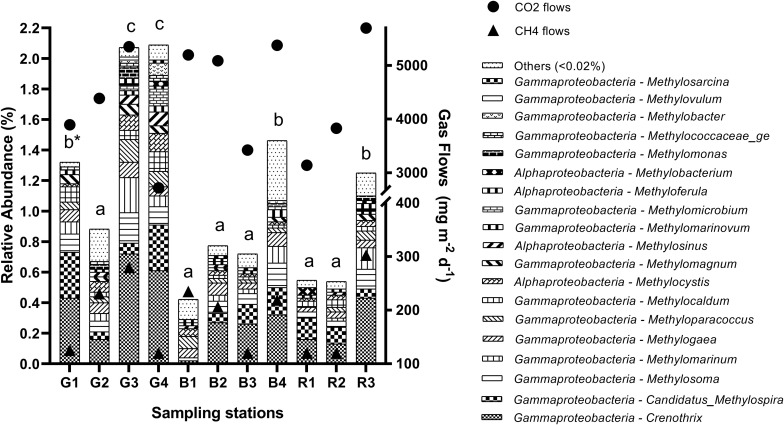
Relative abundance of aerobic methanotrophic genera in the sediment of reservoirs and the atmospheric CO_2_ and CH_4_ flows throughout the sampling stations. “Others” refers to a group of genera with abundances lower than 0.02%. *Means with equal letters do not differ by the Scott–Knott test at 5% significance to methanotrophs abundance.

Several aerobic methanotrophic genera (abundance > 0.02%) were found in the sediments throughout the seasons (Figure 4) and stations (Figure 5). Overall, *Crenothrix* spp. was the most abundant aerobic methanotroph, followed by *Candidatus* Methylospira spp. in some stations, both from the *Gammaproteobacteria* class.

As observed for methanogenic genera, the relative abundance of the total aerobic methanotrophic community was similar throughout the seasons (Figure 4) and differed spatially (*p* = 0.0011), being higher especially at G3 (2.06%) and G4 (2.08%), but also in G1 (1.32%), B4 (1.49%), and R3 (1.24%) (Figure 5). For the gaseous flows, G3, B4, and R3 were among the sites with high CH_4_ (279.9 ± 111.3 mg m^–2^ d^–1^, 218.9 ± 82.5 mg m^–2^ d^–1^ and 302.7 ± 97.2 mg m^–2^ d^–1^, respectively) and CO_2_ flows (5,351.30 ± 774.20 mg m^–2^ d^–1^, 5,377.46 ± 1,054.05 mg m^–2^ d^–1^ and 5,697.67 ± 845.79 mg m^–2^ d^–1^, respectively). Considering the spatially differences occurred in the limnological variables analyzed, G1, G3, G4, B4, and R3 were among the sites with high DTC (14.58 ± 2.72 mg L^–1^, 18.60 ± 4.06 mg L^–1^, 13.32 ± 6.79 mg L^–1^, 15.58 ± 2.01 mg L^–1^, and 13.97 ± 3.88 mg L^–1^, respectively). All the differences observed in the other limnological variables did not occur among G1, G3, G4, B4, R3, and the other sampling stations, and did not reflect on the relative abundance of the total methanotrophic community.

In the sediments of the studied reservoirs, no microorganisms known as anaerobic methanotrophic were found, but some groups with potential for this metabolism are discussed forward.

### Microbes Correlated With CH_4_ and CO_2_ Flows

**FIGURE 6:**
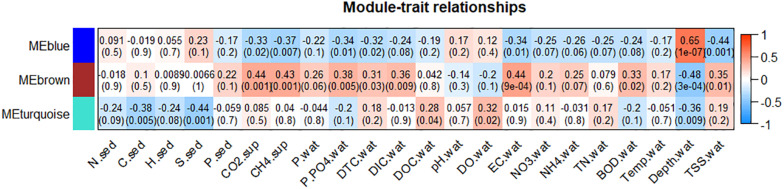
Correlations between eigen genes modules and limnological variables. sed, sediment; wat, water; sup, air-water interface.

During WGCNA clustering, the sample corresponding to the G3 station from autumn was removed as an outlier. The 358 genera of microorganisms included in WGCNA were grouped into three modules, namely blue, brown, and turquoise, encompassing, respectively, 125, 76, and 157 microbial genera. Among these genera, 117 showed correlation with CH_4_ flows, while 121 correlated with CO_2_ flows (Supplementary Table 3). Crossing module correlations with limnological variables (Figure 6), the brown module was positively correlated with both CO_2_ and CH_4_ (*p* = 0.001), besides P-PO_4_^3–^ (*p* = 0.005), DTC (*p* = 0.03), DIC (*p* = 0.009), EC (*p* = 9e-04), BOD (*p* = 0.02), and TSS (*p* = 0.01), and negatively correlated to depth (*p* = 3e-04). The blue module was negatively correlated with CO_2_ (*p* = 0.02) and CH_4_ (*p* = 0.007), as well as P-PO_4_^3–^ (*p* = 0.01), DTC (*p* = 0.02), EC (*p* = 0.01), and TSS (*p* = 0.001), and positively correlated with depth (*p* = 1e-07). Finally, the turquoise module did not show correlations with gaseous flows and was not discussed.

Some methanogenic microorganisms were not significantly correlated with CH_4_ flows (Supplementary Table 3), including *Methanolinea* (*p* = 0.7546), *Methanoregula* spp. (*p* = 0.1426), *SDB* (*Methanomicrobia*) (*p* = 0.4890), *uncultured Methanomicrobia* (*p* = 0.4224), and *uncultured Thermoplasmata* (*p* = 0.2798). Additionally, *Methanosaeta* (*p* = 0.0203), *Methermicoccus* spp. (*p* = 0.0257), and *Methanomicrobiaceae_ge* (class *Methanomicrobia*) (*p* = 0.0195) presented negative correlations with CH_4_ flows.

Regarding known aerobic methanotrophs, none presented positive correlation with CH_4_ flows, although *Candidatus* Methylospira spp. (*Gammaproteobacteria*) was negatively correlated to this gas (*p* = 0.0320). Additionally, several methanotrophic genera were not correlated with CH_4_, including *Methylomarinum* (*p* = 0.1224), *Methylomarinovum* (*p* = 0.1657), *Methylosoma* (*p* = 0.1658), *Methylomagnum* (*p* = 0.1829), *Methylogaea* (*p* = 0.2393), *Methylocaldum* (*p* = 0.4533), *Crenothrix* (*p* = 0.6185) (*Gammaproteobacteria*), and *Methylocystis* spp. (*p* = 0.2700) (*Alphaproteobacteria*) (Supplementary Table 3).

**FIGURE 7:**
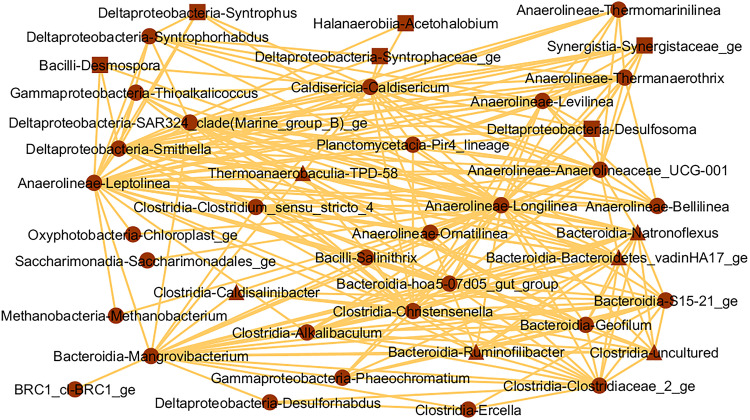
Interaction network of microbial genera in the brown module (threshold = 0.15), which presented positive correlation (*p* ≤ 0.05) with CH_4_ (triangles), CO_2_ (squares), or both gases (circles).

**FIGURE 8:**
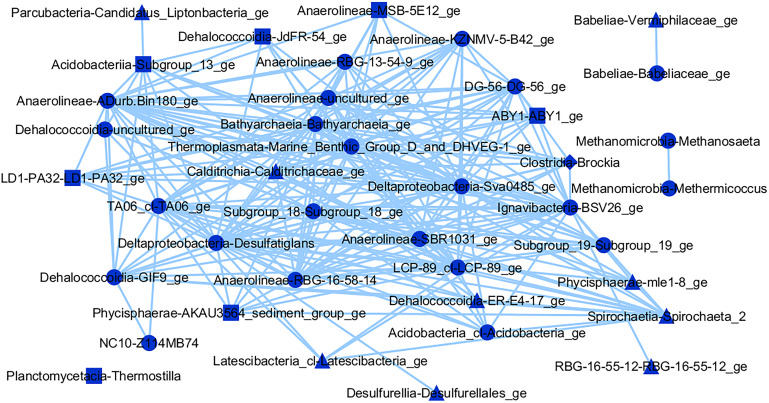
Interaction network of microbial genera in the blue module (threshold = 0.15), which presented positive correlation (*p* ≤ 0.05) with CH_4_ (triangles), CO_2_ (squares), or both gases (circles).

Analyzing the microorganism interaction network of the brown module (Figure 7), *Methanobacterium* spp. was the only methanogenic archaea observed, and interacted directly with the fermenters *Leptolinea* and *Longilinea* spp. No known aerobic methanotrophs were observed in this module. In the blue module microorganisms’ interaction network (Figure 8), two known methanogenic archaea from the *Methanomicrobia* class were identified, namely *Methermicoccus* and *Methanosaeta* spp. However, both microorganisms interacted exclusively with each other, and do not present interactions with the remaining microorganisms of the module, considering the threshold used. Similar to the brown module, no known aerobic methanotroph was observed in the blue module.

## Discussion

### Methanogenic and Aerobic Methanotrophic Communities and Gaseous Flows

In this research we analyzed, seasonally and spatially, the relative abundance of the total methanogenic and aerobic methanotrophic communities in sediments of three impacted urban freshwater reservoirs located in the metropolitan city of São Paulo, SP, Brazil. The identification of several methanogenic genera co-existing in these tropical reservoirs suggests that a variety of substrates are available for methanogenesis (Parro et al., 2019). The acetoclastic *Methanosaeta*, and the hydrogenotrophs *Methanoregula* and *Methanolinea* spp. were the dominant methanogenic genera detected and may represent up to 60% of environmental 16S rRNA sequences in freshwater lakes (Borrel et al., 2011). *Methanoregula* and *Methanolinea* spp. have already been noted with high abundance in the bottom water column of a subtropical freshwater reservoir in Australia (Musenze et al., 2016).

*Methanosarcina* spp. and relatives in the *Methanosarcinales* order, common in such freshwater sediments (Mach et al., 2015; Chaudhary et al., 2017), were not observed in high abundance in sediments of these reservoirs, similar to that found in the water column of a subtropical reservoir in Australia (Musenze et al., 2016). The higher abundance of *Methanosaeta* and lower abundance of *Methanosarcina* spp. may indicate low acetate concentrations in these sediments (Jetten et al., 1992; Smith and Ingram-Smith, 2007). Despite *Methanosaeta* spp. being the most abundant methanogenic in all seasons and stations (Figures 2, 3), the cumulative abundance of all hydrogenotrophic genera surpasses the acetoclastic ones, suggesting hydrogen as an equally important substrate for methanogenesis in tropical reservoirs, which seems common in freshwater sediments (Mach et al., 2015; Gruca-Rokosz and Koszelnik, 2018).

In the reservoirs, the temperature did not affect the relative abundance of the total methanogenic community. However, in a subtropical freshwater environment, methanogenic abundance increased following increase in temperatures higher than 27°C in rainy seasons, while abundance of this group was lower in dry seasons with temperature varying from 26 to 18°C (Zhang et al., 2020). The range of temperature in the studied reservoirs was 25–19.4°C and probably was not wide enough to influence the methanogenic group abundance. Additionally, temperature did not seem to influence the gas flow, as already noted, suggesting more influence of organic matter composition (not evaluated here) in this environment (Gruca-Rokosz and Koszelnik, 2018). Furthermore, differences in all the other limnological variables throughout the seasons did not affect the gaseous flows or the abundance of methanogenic and methanotrophic groups.

The greater depth in some sites of the studied reservoirs enhanced methanogenic abundance but decreased gas flows. In fact, shallower sampling stations suggest that the ebullitive process (gas bubbles) may have contributed to increasing CH_4_ flows in these stations (Capone and Kiene, 1988). Conversely, in deeper sites, besides the rarity of this ebullitive process (West et al., 2016), CH_4_ may also have been oxidized in the largest water column (Zigah et al., 2015) by microorganisms not studied in the present research.

Counterintuitively, the more abundant methanogenic community in sediments, including those in B3 in Billings, previously characterized with low pollution, did not show higher CH_4_ flows in the reservoirs. It can be explained by the fact that methanogenesis potential in freshwater sediments may be influenced by the activity of other microbial community members or by substrate availability, besides the presence of methanogenic microorganisms (Chaudhary et al., 2017). Microbial enrichment experiments with sediments from a reservoir in Poland corroborate to higher CH_4_ production with a reduced proportion of methanogens (Szafranek-Nakonieczna et al., 2019), suggesting uncoupling of abundance and activity. This is observed in our study for the first time in field samples of tropical freshwater reservoirs. In fact, the non-methanogenic microbial community in freshwater environments greatly influence this bioprocess, since they provide the substrates involved in methanogenesis (Bertolet et al., 2019), compete for these substrates with methanogens and/or may oxidize CH_4_. For instance, methanogenesis is known to be inhibited by a limitation of substrates when competing with nitrate, iron or sulfate reducers (Klüber and Conrad, 1998; Roden and Wetzel, 2003; Bae et al., 2015).

Regarding aerobic methanotrophic community and limnological variables, in the studied reservoirs, total carbon of bottom water seems to affect the abundance of these microorganisms in sediments, which has already been reported in paddy soil (Tian et al., 2020). The most abundant methanotroph detected in the studied reservoirs, *Crenothrix* spp. has been reported as an important CH_4_ consumer in freshwater environments (Oswald et al., 2017) and was already identified with high abundance in the water column of a subtropical freshwater reservoir in Australia (Musenze et al., 2016). Nevertheless, our results also suggest that higher known relative abundance of the total methanotrophic community in sediments does not reflect decreased CH_4_ flows, similar to previous observations regarding abundance and diversity of this community in soils (Li et al., 2020). Moreover, given the widespread role of this group in reducing CH_4_ flows in freshwater environments and wetlands (Segarra et al., 2015; Zigah et al., 2015; Martinez-Cruz et al., 2018), we suggest that CH_4_ flows in the studied reservoirs would be higher without the presence of methanotrophic microorganisms.

### Sediment Microbial Communities Acting in Water-Air Gaseous Flows

We identified the key microbial players in sediment involved in atmospheric CH_4_ and CO_2_ flows in three tropical urban freshwater reservoirs. Most methanotrophs and methanogens did not correlate with CH_4_ flows. The brown interaction network (Figure 7) offers insightful glimpses into the community positively correlated to CH_4_ and CO_2_ flows. We observed the hydrogenotrophic methanogen *Methanobacterium* spp., positively correlated with CH_4_ and alleged interaction with fermenters *Leptolinea* and *Longilinea* spp., which can provide their substrates (Yamada et al., 2006, 2007). *Longilinea* spp. was already shown to improve growth in co-culture with hydrogenotrophic methanogens (Yamada et al., 2007). In the same interaction network, fifteen more fermenter genera could also provide substrates for methanogenesis indirectly, including *Levilinea*, *Bellilinea*, *Syntrophus*, and *Smithella* spp. (Mountfort et al., 1984; Liu et al., 1999; Yamada et al., 2006, 2007). Six other potential fermenters, given molecular data or phylogenetic similarity, were also detected including *BRC1* and *Pir4_lineage* (Kadnikov et al., 2019; Dedysh et al., 2020). This fermenter community diversity strongly suggests that plenty of fermentative subproducts are available and indicates the occurrence of syntrophic associations. In fact, the *Anaerolineae* class may form syntrophic cooperation with methanogens (Liang et al., 2015), which were also observed for *UCG-001* (de Leeuw et al., 2019). Similarly, *Syntrophorhabdus*, *Syntrophus*, and *Smithella* spp., from the *Deltaproteobacteria* class, and *Caldisericum* spp., from *Caldisericia* class, present in the same brown network, have demonstrated this syntrophic potential previously (Mountfort et al., 1984; Guyot and Brauman, 1986; Liu et al., 1999; Qiu et al., 2008; Szafranek-Nakonieczna et al., 2019), although a direct interaction with *Methanobacterium* spp. was not detected here. No known methanotroph was highlighted in the brown module interaction network.

On the other hand, the blue interaction network (Figure 8) gives a novel insight into a community inversely correlated with CH_4_ and CO_2_ flows. Two methanogens were observed among the microorganisms in this network, the methylotrophic *Methermicoccus* and the acetoclastic *Methanosaeta* spp. It is theorized that *Methanosaeta* spp. is also capable of CH_4_ oxidation, given its phylogenetic similarity to anaerobic methanotrophs (Smith and Ingram-Smith, 2007), which could explain its direct link to the former. Many other microbes have not been characterized in this module, hindering metabolic inferences of their role in the environment. An exception is *Thermostilla* spp. which is capable of producing H_2_, acetate, and CO_2_ through fermentation (Slobodkina et al., 2016). Nevertheless, there was no direct interaction of this group with any microbes correlated with gases in the blue module. Probably at least eleven groups, including seven from the *Anaerolinea* class, are also fermenters, given that all previously described members of this class present such metabolism (Narihiro et al., 2012), but no corroborative studies were found to support such inferences in these cases.

Although no aerobic methanotrophs were observed in the blue interaction network, several probable anaerobic CH_4_ oxidizers were found within this module. The *Sva0485* group (*Candidatus* Acidulodesulfobacterales) has been shown to participate in anaerobic CH_4_ oxidation, in an unspecified role (Bar-Or et al., 2015), and can reduce sulfate or iron (Tan et al., 2019). Within the *NC10* class, a denitrifying methanotroph was recently identified, coupling nitrate reduction to CH_4_ oxidation (Versantvoort et al., 2018), and this group has been observed in other freshwater environments (Graf et al., 2018). In the *Thermoplasmata* class, the *MBG-D/DHVEG-1* group has been observed in anaerobic CH_4_ oxidation zones, also in freshwater environments (Schubert et al., 2011), possibly coupling iron reduction to CH_4_ oxidation (Bar-Or et al., 2015). The phylum *Candidatus* Bathyarchaeota was reported as capable of coupling reverse methanogenesis with denitrification (Harris et al., 2018).

Additionally, several microbes involved in the reduction of inorganic compounds are also present, which could receive electrons from the oxidation of H_2_ or CH_4_. Some genera, including *Brockia*, *Desulfatiglans*, and *Thermostilla* spp. are known to reduce sulfur, sulfate and/or nitrate (Perevalova et al., 2013; Suzuki et al., 2014; Slobodkina et al., 2016). At least eight other groups, through phylogenetic similarity or genomic analyses, could be inferred to participate in similar processes, including *LCP-89* (Huang et al., 2019). Nevertheless, no direct link with a methanogen and other metabolic groups was observed in the blue module interaction network.

Since microbial community composition seems one of the most important factors for CH_4_ production and emission (Liebner et al., 2015; Chaudhary et al., 2017; Bertolet et al., 2019), comparing both communities discussed above can improve such understanding in tropical urban reservoirs. Fermentation was clearly the dominant metabolism in the first community (brown), which may explain its positive correlation with CH_4_ flow, despite the low abundance of methanogens. This implies that the conversion of organic compounds into simpler substrates (including H_2_ and acetate) used by methanogens is more important to gas formation (Bertolet et al., 2019). Additionally, many fermenters also produce CO_2_, contributing directly to its flows and to the positive correlation observed. The second community (blue) also presented its share of potential fermenters, but most of them are not empirically determined, and therefore it is not possible to infer their subproducts, which directly influence methanogenesis (Uz and Ogram, 2006). Furthermore, both known methanogens present in this network, *Methermicoccus* and *Methanosaeta* spp., did not show direct co-occurrence with other microbes, suggesting weak to no interaction. Finally, possible anaerobic CH_4_ oxidizers or inorganic compound reducers were observed in the second community, likely promoting increased CH_4_ consumption (Segarra et al., 2015; Martinez-Cruz et al., 2018) and enhancing competition for substrates between methanogens and other anaerobic respiration heterotrophs (Klüber and Conrad, 1998; Roden and Wetzel, 2003; Bae et al., 2015). All of these results would contribute to lower flows of CH_4_, as observed in the blue community.

### Limnological Factors Affecting Communities Correlated With Gaseous Flows

A direct correlation between the CH_4_ and CO_2_ emitting community and limnological parameters known as pollution indicators in reservoirs was observed, including orthophosphate, organic matter (DTC and BOD), EC and TSS, and an inverse correlation of these variables with the community not favoring CH_4_ and CO_2_ flows (Figure 6). These results corroborate that pollution favors gaseous flows in water bodies (Gonzalez-Valencia et al., 2014; Silva et al., 2016), by supporting the microbial community acting in these flows, and reinforce the concept that management measures, aiming at preventing or reducing anthropogenic impacts, should also be considered to mitigate CH_4_ and CO_2_ emissions from reservoirs (Ometto et al., 2013). Domestic sewage discharges are the likely impact on these reservoirs, according to the values of P-PO_4_^3–^, EC, DTC, TSS, and BOD, and should be prevented or at least minimized (Shiddamallayya and Pratima, 2008; Tappin et al., 2016; Vigiak et al., 2019; Coelho et al., 2020). Although not innovative, such observations in environmental samples reinforce the robustness of our data and strengthen the remainder conclusions drawn in this research.

The bioprocesses involved in CH_4_ and CO_2_ emissions are directly affected by all these factors. P-PO_4_^3–^ influences methanogenesis and upstream metabolic pathways, including acidogenesis and acetogenesis (Wang et al., 2015). DTC availability is a likely determinant of maximum CO_2_ and CH_4_ production (Mulka et al., 2016), while BOD also implies organic carbon availability (Vigiak et al., 2019). Higher DIC concentration in aquatic environments originate from organic carbon remineralization to CO_2_ and methanogenesis (Ogrinc et al., 2002), corroborated by the positive correlation observed. Higher EC strengthens direct electron transfer between syntrophs and methanogens, also increasing CO_2_ reduction to CH_4_ during hydrogenotrophic methanogenesis (Ye et al., 2018). Fermenters from the *Anaerolineae* class, which interacted directly with *Methanobacterium spp.* in the gaseous emitting community, were recently suggested to directly transfer electrons to other microbes (Zhuang et al., 2019). Conversely, this process would be hindered in conditions favoring the community correlated with decreased CH_4_ flows. Shallower water bodies, such as those observed in this study, are probable sources of increased gaseous emission by sediments (Peeters et al., 2019), since most of the organic carbon is converted into CH_4_ and liberated actively to the atmosphere through an ebullitive process (Capone and Kiene, 1988; West et al., 2016). The present study complements that a shallower depth also seems to contribute to a higher abundance of the microbial community which produces CH_4_ and CO_2_, while microbes that promote decreased gaseous flows are favored in deeper regions.

## Conclusion

In the eutrophic tropical reservoirs studied, depth and DTC in bottom water influenced, respectively, the relative abundance of the total methanogenic and aerobic methanotrophic communities in the sediments. Several methanogens that produce CH_4_ mainly by the acetoclastic and hydrogenotrophic pathways were identified in the sediments, but the overall relative abundance of this group did not promote the higher atmospheric CH_4_ flows. Similarly, the relative abundance of the total aerobic methanotrophs in the sediments did not reflect the lower flows of this gas, supporting the uncoupling between abundance and activity. Anthropogenic pollution indicators, including BOD and orthophosphate, favored fermentative microorganisms, converting organic matter into methanogenesis substrates, which were directly associated with atmospheric CH_4_ and CO_2_ flows. Mitigation of these parameters allowed the development of a microbial community with probable anaerobic CH_4_ oxidizers, capable of coupled processes as inorganic compounds (sulfate, nitrate, and iron) reduction, despite the lack of available information for several microbe components. These results corroborate that sanitation measures can potentially reduce gaseous emissions in eutrophic tropical urban reservoirs, which could mitigate the warming impact and favor biotransformation of inorganic compounds in these freshwater environments. Further research is needed to evaluate the active microorganisms in the water column, which are also involved in gas production and consumption and take new metabolic characterizations of uncultured microorganisms through more advanced molecular techniques in enrichments of these freshwater sediments, to increase the knowledge about microbial role in gas flows in reservoirs.

## Data Availability Statement

The datasets presented in this study can be found in online repositories. The names of the repository/repositories and accession number(s) can be found below: https://www.ncbi.nlm.nih.gov/, SRA SRP301026, BioProjectID PRJNA690112, experiments SRX9810531–SRX9810580.

## Author Contributions

GG, FS, MD, and RB designed the study. TJ, LC, WH, MP, MD, and RB were responsible for field work, limnological variables, and gas flow measurements. GP and GG were responsible for microbial lab work and data integration, with support from MD and FS. GG, GP, MD, FS, and RB wrote the initial manuscript. All authors read and contributed to the final manuscript, being accountable for its content.

## Conflict of Interest

The authors declare that the research was conducted in the absence of any commercial or financial relationships that could be construed as a potential conflict of interest.
