# Corrigendum: Higher Abundance of Sediment Methanogens and Methanotrophs Do Not Predict the Atmospheric Methane and Carbon Dioxide Flows in Eutrophic Tropical Freshwater Reservoirs

**DOI:** 10.3389/fmicb.2021.822946

**Published:** 2021-12-16

**Authors:** Gabrielle Maria Fonseca Pierangeli, Mercia Regina Domingues, Tatiane Araujo de Jesus, Lúcia Helena Gomes Coelho, Werner Siegfried Hanisch, Marcelo Luiz Martins Pompêo, Flávia Talarico Saia, Gustavo Bueno Gregoracci, Roseli Frederigi Benassi

**Affiliations:** ^1^Institute of Marine Sciences, Federal University of São Paulo, Santos, Brazil; ^2^Center of Engineering, Modeling and Applied Social Sciences, Federal University of ABC, Santo André, Brazil; ^3^Chemical Engineering Department, Federal University of São Paulo, Diadema, Brazil; ^4^Ecology Department, State University of São Paulo, São Paulo, Brazil

**Keywords:** greenhouse gases, sediment microbiota, metagenomics, anthropic pollution, network analysis

In the original article, we neglected to include the funder São Paulo Research Foundation (FAPESP), process number 2021/01547-0 to Gustavo Bueno Gregoracci. The Funding statement has been updated. The corrected paragraph appears below:

“This study was supported by the São Paulo Research Foundation (FAPESP–process number: 2017/10355-1), with technical training scholarship (process number: 2017/19001-8; 2018/20417-7; 2019/23767-1), and by the Brazilian National Research Council (CNPq), with master's scholarship (process number: 167185/2018-7). The publication fee was supported by the São Paulo Research Foundation (FAPESP–process number 2021/01547-0).”

The authors apologize for this error and state that this does not change the scientific conclusions of the article in any way. The original article has been updated.

## Publisher's Note

All claims expressed in this article are solely those of the authors and do not necessarily represent those of their affiliated organizations, or those of the publisher, the editors and the reviewers. Any product that may be evaluated in this article, or claim that may be made by its manufacturer, is not guaranteed or endorsed by the publisher.

